# Ordering Dynamics in Neuron Activity Pattern Model: An Insight to Brain Functionality

**DOI:** 10.1371/journal.pone.0141463

**Published:** 2015-10-27

**Authors:** Jasleen Gundh, Awaneesh Singh, R. K. Brojen Singh

**Affiliations:** 1 School of Computational and Integrative Sciences, Jawaharlal Nehru University, New Delhi-110067, India; 2 School of Physical Sciences, Jawaharlal Nehru University, New Delhi–110067, India; Universiteit Gent, BELGIUM

## Abstract

We study the domain ordering kinetics in *d* = 2 ferromagnets which corresponds to populated neuron activities with both long-ranged interactions, *V*(*r*) ∼ *r* ^−*n*^ and short-ranged interactions. We present the results from comprehensive Monte Carlo (MC) simulations for the nonconserved Ising model with *n* ≥ 2, interaction range considering near and far neighbors. Our model results could represent the long-ranged neuron kinetics (*n* ≤ 4) in consistent with the same dynamical behaviour of short-ranged case (*n* ≥ 4) at far below and near criticality. We found that emergence of fast and slow kinetics of long and short ranged case could imitate the formation of connections among near and distant neurons. The calculated characteristic length scale in long-ranged interaction is found to be *n* independent (*L*(*t*) ∼ *t* ^1/(*n*−2)^), whereas short-ranged interaction follows *L*(*t*) ∼ *t* ^1/2^ law and approximately preserve universality in domain kinetics. Further, we did the comparative study of phase ordering near the critical temperature which follows different behaviours of domain ordering near and far critical temperature but follows universal scaling law.

## Introduction

Brain is a complex system that works through an interplay of neurons. The spiking activity in complex neuron network in brain is dynamic (far from equilibrium) [1]. It has been modeled as a network of neurons to mimic brain dynamics using Ising model and found that neural population exhibit nonequilibrium critical dynamics [2] and the criticality in it has been used to characterize brain signals [3]. The reason could be when such system starts to be quenched from a disordered phase to an ordered phase, it becomes thermodynamically unstable. The subsequent *far-from-equilibrium* evolution of the system is characterized by the emergence and growth of domains enriched in the new equilibrium phases. This nonequilibrium evolution, usually called *kinetics of phase ordering* or *domain growth*, has been the subject of much active investigation [4]. The domain here represents an ordered set of active/inactive neurons. The domain morphology is quantified by the time dependence of the domain scale *L*(*t*), where *t* is the time after the quench. There is a good understanding of domain growth kinetics in pure and isotropic systems with short-ranged interactions, where the domain scale shows a power-law behavior, *L*(*t*) ∼ *t* ^*ϕ*^ [5, 6]. For the case with nonconserved order parameter, e.g., ordering of a ferromagnet into up and down phases (spin-flip Glauber-Ising model [7]), one has *ϕ* = 1/2 [8, 9]. On the other hand, for the case with conserved order parameter, e.g., phase separation of a binary (*AB*) mixture into *A*- and *B*-rich domains (spin-exchange Kawasaki-Ising model [10]), we have *ϕ* = 1/3 when growth is driven by diffusion [11, 12]. Apart from the domain growth laws, experimentalists are also interested in quantitative features of the domain morphologies. An important experimental quantity is the time-dependent correlation function $C(\vec{r},t)$ that measures the correlation of a spin (neuron) with the rest of the spins with time, or its Fourier transform, structure factor $S(\vec{k},t)$ ($\vec{k}$ being the wave vector) [4, 5]. Most of the studies are concentrated around the nearest-neighbor (short-range) inter-molecular interaction.

Ferromagnetic ising system is pioneer yet simple equilibrium model that has been used extensively to understand brain dynamics [13, 14]. However, analogy of this model with brain dynamics sounds skeptical [15], but some empirical results of neuron spiking activity from network of neurons has shown phase transition from low to high activity at criticality [16–19] with optimum information transmission [20, 21] Here, we have used the concept of phase ordering dynamics in Ising model as an effort to study brain functionality reflected from long and short range interaction of neurons. We need to target how brain executes a specific task and forms different patterns each time for a specific activity [22]. Multiple patterns are being formed with both long and short range interactions. The neuron activities in brain at critical point are believed to be effective for the long distance communication of the neurons [23] because of coupling and variability to optimize information storage in the system [24] and dynamic range of the system to response the input signal [25]. The core of the paper focuses on long-range interactions which dynamically explains the rapid movement of the signal information inside the brain. The fast emergence of long-range interactions, mimic the rapid neuronal interactions in the brain. Further, we study the phase ordering dynamics in neurons with a specific interest to understand the role of the range of inter-neuron interactions. We address two important questions in this context via kinetic MC simulations: (a) What is the growth law for ordering phases of neurons? Is the growth law independent of the range of interaction? (b) What is the morphology for ordering phases of neurons, as measured by the correlation function and structure factor? Is it comparable for all interaction range? We will be providing the answers to the above questions from our extensive MC simulations.

## Neuron Activity Pattern Model

Brain can be considered as a complex network of neurons. In brain, network rewiring of hundred billion neurons, forming multiple patches/patterns of firing neurons exhibiting a specific cognitive function is the crust of brain functioning. In an effort to understand the gist of pattern formation, we can think of brain as a system (square lattice) with neurons as number of particles which can be mapped onto ferromagnetic Ising model with two spin interactions [26], where random firing or non-firing of neuron can be represented by two states of a spin, *s* = +1 for firing and *s* = −1 for rest or non-firing neurons [27]. Even though neuron activity pattern model is far from equilibrium dynamic model [18], Ising model can be serve an excellent model to deal with critical phenomena of neuron activity pattern [1]. The large number of local (short range) interaction of neurons [28] and significant amount of global (long range) interaction of neurons [29] are main basis of neuronal communication in brain network [30]. The transition from a disordered system of neurons to an ordered domain is studied using the long-ranged spin (neuron) model (LSM). We consider the following Hamiltonian of two dimensional Ising system which incorporates the (LSM),

$$H=-\sum_{<ij>}J\left(r_{ij},n\right)s_{i}s_{j},\;s_{i}=\pm 1,$$ (1)

where *J* is the coupling strength, *n* characterizes the range of the interaction, $r_{ij}=\left|{\vec{r}}_{i}-{\vec{r}}_{j}\right|$, and *s*_*i*_ denotes the spin variable at site *i*. We consider two state spins: *s*_*i*_ = +1 denotes an up-spin (active neurons) and *s*_*i*_ = −1 denotes a down-spin (inactive neurons). We consider only a ferromagnetic case, where *J* > 0 always. The case where *J* can be both > 0 (ferromagnetic) and < 0 (antiferromagnetic) is relevant to spin glasses. We associate stochastic dynamics with the Ising model by placing it in contact with a heat bath. The appropriate dynamics for the phase ordering problem is *spin-flip kinetics* or *Glauber kinetics*.

If we consider interacting neurons (spins) through slowly decay potentials [31]. In order to capture thermodynamical parameters, one can define the following potential function (obeying power law functional form) [32, 33],

$$\begin{array}{c} U\left(n\right)=\lim_{N\to \infty }\frac{1}{N}\sum_{i,j;i\neq j}^{N}\frac{J}{r_{ij}^{n}}. \end{array}$$ (2)

Here *n* = *d* + *σ* = 2 + *σ* [34, 35] for two dimensional system. For short-ranged interaction, *σ* > 2 and the system size is not much important, whereas for long-ranged interaction, 0 < *σ* < 2 and it depends on the system size [36, 37]. The functional form of *J*(*r*_*ij*_, *n*) is given by $J\left(r_{ij},n\right)=J/r_{ij}^{n}$, where, *J* > 0 and *n* > 0 [33]. The asymptotic values of *J* are, for *n* → ∞ corresponds to nearest neighbour, and *n* → 0 with *J* → *J*/*N* corresponds to Currie-Weiss model [33]. Since, the size of the system is *N*, rescaling *J* → *J*/*N* to the Curie-Weiss model [33] and using Euler-McLaurin sum formula [38] for *N* ≫ 1, Eq (2) can be written as,

$$\begin{array}{ll} U(n) & =\underset{N\to \infty }{\text{lim}}\;J\;\sum_{x=1}^{\sqrt{N}}\sum_{y=1}^{\sqrt{N}}\frac{1}{{\left(x^{2}+y^{2}\right)}^{(n-2)/2}},\\ & \approx \underset{N\to \infty }{\text{lim}}\;J^{d(=2)}\;\int_{1}^{\sqrt{N}}drg(r)r^{3-n}, \end{array}$$ (3)

where, *g*(*r*) is the pair distribution function such that *g*(*r*) ≈ 1 for *r* ≫ 1. The integration in Eq (3) can be evaluated considering three conditions of *n*: (**a**) **n** = **4**, the Eq (3) becomes, $U\left(n\right)\approx J\;\lim_{N\to \infty }\int_{1}^{\sqrt{N}}\frac{dr}{r}\approx \frac{J}{2}\;\lim_{N\to \infty }\ln\left(N\right)$, (**b**) **n** **>** **4**, the Eq (3) can be written as, $U\left(n\right)\approx J\;\lim_{N\to \infty }\int_{1}^{\sqrt{N}}drr^{-(n-3)}\approx \frac{J}{n-4}\;\lim_{N\to \infty }[1-N^{2-n/2}]$, and (**c**) **0** **<** **n** **<** **4**, the Eq (3) becomes, $U\left(n\right)\approx J\;\lim_{N\to \infty }\int_{1}^{\sqrt{N}}drr^{3-n)}\approx \frac{J}{4-n}\;\lim_{N\to \infty }N^{2-n/2}$. Combining all three cases, we can reach the following equation,

$$\begin{array}{c} U\left(n\right)\approx \lim_{N\to \infty }J\;[\begin{array}{ccc} \frac{1}{2}\ln\left(N\right) & \text{for} & n=4,\\ \frac{1}{n-4}\;(1-N^{2-n/2}) & \text{for} & n>4,\\ \frac{1}{4-n}N^{2-n/2} & \text{for} & 0<n<4. \end{array}] \end{array}$$ (4)

The existence of critical point of the force derived from the potential Eq (2) depends on the nature of the force (positive or negative) due to which singularity arises in the solution of the system [32]. However, closed form approximation in the numerical solution of the system was used by Hiley and Joyce [32] and could able to estimate critical point at which one can predict critical thermodynamical parameters, as given below,

$$\begin{array}{c} \frac{U(n)}{k_{B}T_{C}}=1+\frac{f_{2}}{U{\left(n\right)}^{2}}+O\left(U{\left(n\right)}^{-4}\right) \end{array}$$ (5)

where, the *f*_2_ = ∑_*i*,*j*_ *J*(*r*_*ij*_, *n*)^2^ which has to be evaluated for the whole 2D lattice points. For various types of lattice structure, the numerical solution of Eq (5) at large limit of *N* converges approximately to 1 [32], from which critical temparature, *T*_*C*_ can be obtained as follows,

$$\begin{array}{c} T_{C}\approx \frac{J}{k_{B}}U\left(n\right) \end{array}$$ (6)

From Eq (4), one can see that *U*(*n*) is finite for *n* > 4 when *N* → ∞, and the asymptotic behaviour of finite critical temperature *T*_*c*_ [32],

$$\begin{array}{c} T_{c}\left(n\right)\approx \frac{J}{k_{B}}U\left(n\right)\approx \frac{J}{k_{B}}\;(\frac{1}{n-4}), \end{array}$$ (7)

where, *k*_*B*_ is Boltzmann constant. This shows that *T*_*c*_(*n*) ∝ 1/*n* for short-ranged potential (*n* > > 4), whereas for long-ranged potential, *T*_*c*_ depends on the size of the system *N* as well as *n* given by,

$$\begin{array}{c} T_{c}\left(n,N\right)\approx \frac{J}{k_{B}}\;(\frac{1}{4-n})\;N^{2-n/2} \end{array}$$ (8)

and *T*_*c*_ diverges with system size. Further, *T*_*c*_ for *n* = 4 is independent of *n* and is given by,

$$\begin{array}{c} T_{c}\left(N\right)\approx \frac{J}{2k_{B}}\ln\left(N\right) \end{array}$$ (9)

In this case *T*_*c*_ diverges logarithmically with system size *N*. Similarly, one can also calculate other thermodynamical parameters such as internal energy, entropy, free energy per particle (neuron) etc. at this asymptotic limit.

## Details of Simulation

Since it is very difficult to obtain exact analytical solution of this problem, we straightforward implement a MC simulation of the Ising model with spin-flip kinetics to understand the behaviour. In a single step of MC dynamics, we choose a spin at random in the lattice of distribution of spins. The change in energy Δ*H* that would occur if the spin was flipped is computed with the step of acceptance or rejection based on Metropolis acceptance probability [39, 40] given by,

$$\begin{array}{c} P=\{\begin{array}{cc} \exp(-\beta \Delta H) & \text{if}\;\Delta H⩾0,\\ 1 & \text{if}\;\Delta H⩾0. \end{array} \end{array}$$ (10)

where, *β* = (*k*_*B*_ *T*)^−1^ denotes the inverse temperature. One Monte Carlo step (MCS) is completed when this algorithm is performed *N* times (where *N* is the total number of spins), regardless of whether the move is accepted or rejected. All our simulations have been performed on a *d* = 2 lattice of size $L_{s}^{2}$ (*L*_*s*_ = 512) with periodic boundary conditions in both directions. The statistical quantities presented here (e.g., correlation function, structure factor) are obtained as averages over 10 independent runs. Each run starts with a randomly-mixed state with equal numbers of up (active) and down (inactive) spins (neurons), which corresponds to a mean magnetization *m* = 〈*s*_*i*_〉 = 0.

**Fig 1:**
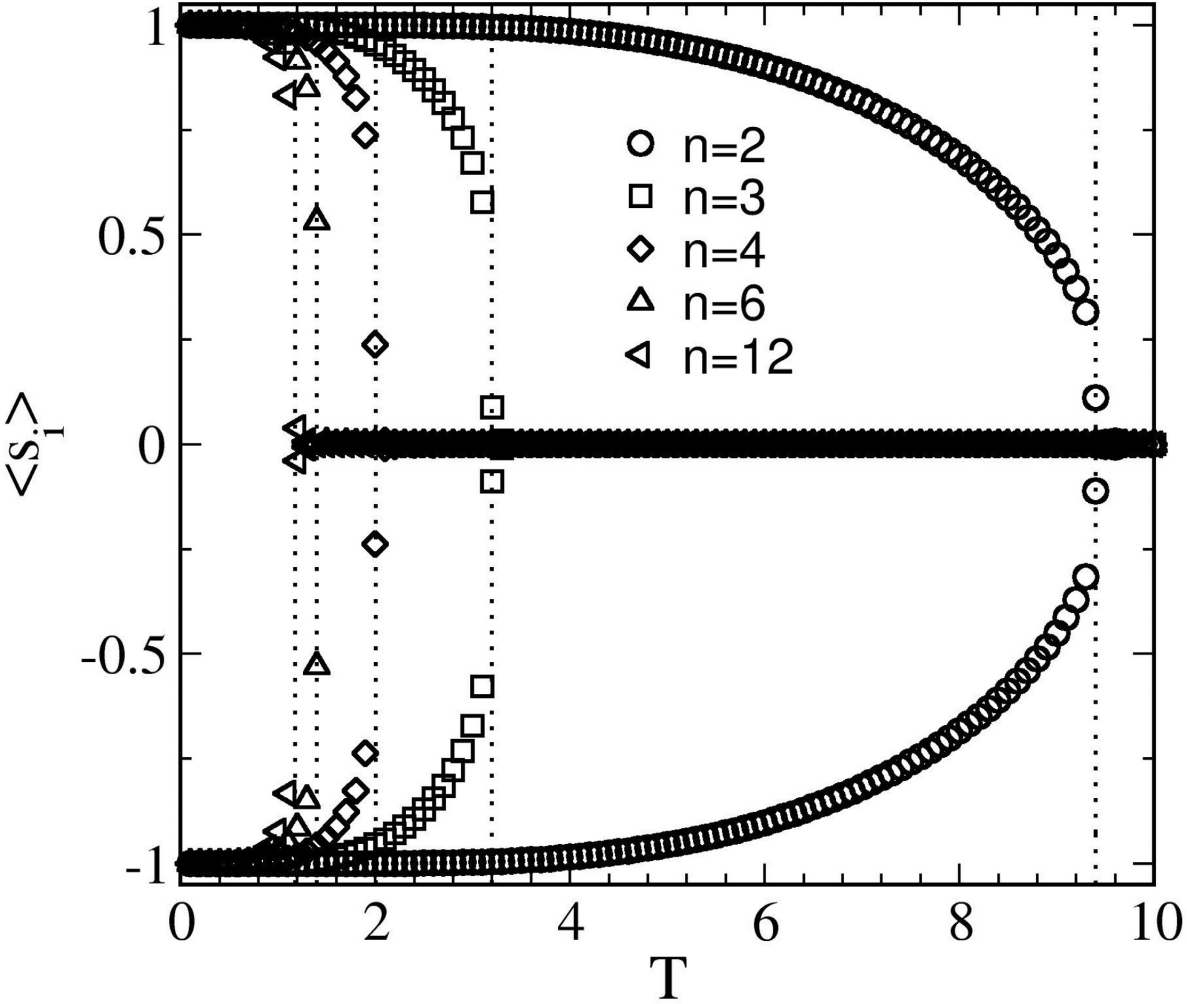
Plot of < *s*_*i*_ > vs. *T* for *n* = 2, 3, 4, 6, and 12 as indicated. The magnetization drops-off sharply near the critical temperature (*T*_*c*_) and then vanishes to 0 in the disordered high-temperature phase.

Here, thinking of the real scenario in the brain, we have considered various interaction ranges (n) that could be taken as multiple synaptic connections in the brain. We study LSM for several values of *n*, namely 2, 3, 4, 6, and 12. The critical ordering temperatures, *T*_*c*_(*n*) for each *n* case, have different points of criticality at which they could mimic brain functionality as shown in Fig 1, where the characteristic behavior of spontaneous magnetization (< *s*_*i*_ >) is plotted against temperature (*T*). As expected, *T*_*c*_(*n*) (dotted lines) increases with decreasing *n* as evident from Eq (7). Above *T*_*c*_ the spontaneous magnetization vanishes, whereas below *T*_*c*_ it takes a nonzero value, inducing the typical behavior of a ferromagnet. Therefore, the physical properties of such systems and so its phase states depend on the value for the magnetization, the parameter which is termed as *order parameter*: an ordered phase in which the spins are aligned appears when *m* ≠ 0, while *m* = 0 implies a disordered (or symmetric) phase. Since, *T*_*c*_’s for *n* ≥ 4 are very close to each-other and hence, exhibit qualitatively similar behavior (explained in next section). We thus consider *n* < 4 cases for the long-ranged interaction. For each value of *n*, we cut-off the interaction at *r*_*c*_ = (2.5)^6/*n*^ to accelerate our simulation [41]. We stress that the simulations are numerically very demanding for larger cut-offs. We compute several statistical quantities to characterize the system. These are described as follows.

The domain coarsening is characterized by a growing time-dependent length scale *L*(*t*), measured at any time instant as the radii of the circle of the total area covered by either type of the spin. The domain morphology i.e a small section of particular size taken at any time instant, does not change with time, apart from a scale factor. For a particular interaction range (n), their is a unique length scale *L*(*t*) depending on time by a constant scaling factor. A direct consequence of the existence of a unique length scale is that the system exhibits a dynamical-scaling in the correlation function and structure factor. We compute the time-dependent correlation function:

$$C({\vec{r}}_{i},{\vec{r}}_{j};t)\equiv \langle s_{i}s_{j}\rangle -\langle s_{i}\rangle \;\langle s_{j}\rangle .$$ (11)

Here, the angular brackets denote an averaging over the independent initial ensemble and different noise realizations. As the system is translationally invariant, the correlation function depends only on $\vec{r}={\vec{r}}_{j}-{\vec{r}}_{i}$:

$$C({\vec{r}}_{i},{\vec{r}}_{j};t)=C({\vec{r}}_{i},{\vec{r}}_{i}+\vec{r};t)=C(\vec{r},t).$$ (12)

Usually most experiments study the structure factor, which is the Fourier transform of the real-space correlation function:

$$S(\vec{k},t)=\int d\vec{r}\;C\;(\vec{r},t)\;e^{i\vec{k}\cdot \vec{r}}.$$ (13)

Since the system is isotropic, we can improve statistics by spherically averaging the correlation function and the structure factor. The corresponding quantities are denoted as *C* (*r*, *t*) and *S* (*k*, *t*), respectively. The correlation function and structure factor obey the dynamical scaling forms:

$$\begin{array}{cc} C\left(r,t\right) & =g\left[r/L\left(t\right)\right],\\ S\left(k,t\right) & =L{\left(t\right)}^{d}f\left[kL\left(t\right)\right]. \end{array}$$ (14)

Here, *g*(*x*) and *f*(*p*) are scaling functions; *r* is the separation between two spatial points; *k* is the magnitude of the wave vector; and *d* is the system dimensionality. The characteristic domain size *L*(*t*) is obtained as the distance over which the correlation function decays to some fraction (say half) of its maximum value [*C*(*r*, *t*) = 1 at *r* = 0]. There are several other suitable definitions for computing *L*(*t*), e.g., first zero-crossing of *C*(*r*, *t*), inverse of the first moment of *S*(*k*, *t*). In the scaling regime, all these definitions differ only by constant multiplicative factors [42–44].

## Numerical Results

**Fig 2:**
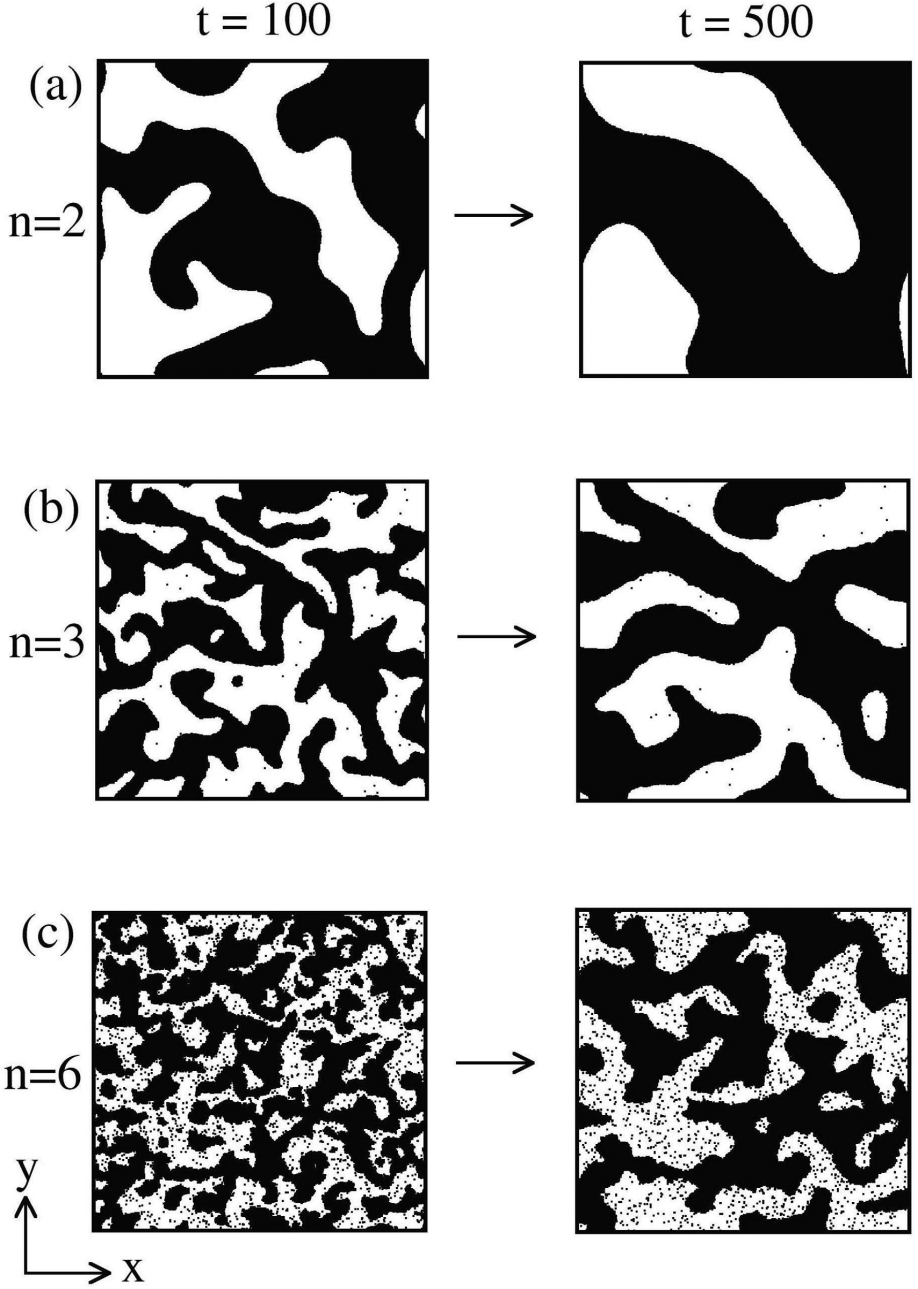
Evolution snapshots of domain coarsening for *n* = 2, 3, and 6 quenched at *T* = 1 below *T*_*c*_ at time *t* = 100, 500. The snapshots are obtained from a Monte Carlo (MC) simulation of ordering kinetics in ferromagnetic system. The details of the MC simulation are provided in the text.

For short-range interaction critical temperature is a function of interaction range only whereas, for long-ranged *T*_*C*_ is a function of both interaction range and system size (see Eqs (4–8)). It is found that short-ranged interactions have their *T*_*C*_s in a narrow spectrum (See Fig 1) thus will have similar coupling strength, however in long-ranged interactions keeping system size constant they have *T*_*C*_ over wide spectrum signifying an exponential change in coupling strength with distance. This could have a direct implication with the synaptic connections made between near and distant neurons in the brain. In Fig 2, we show the evolution snapshots obtained from our MC simulations for *n* = 2, 3, 6 with *T* = 1 (< *T*_*c*_, see Fig 2) at *t* = 100, 500 MCS. At low temperatures, energetic effects are dominant and the system minimizes its energy by ordering the spins parallel to each other. In the absence of an external field (e.g., magnetic field, *h* = 0), the activated neuron (up-spin) and inactivated neuron (down-spin) states are equivalent. In the mean-field (MF) limit, i.e., *n* = 0, all the spins interact with each other and there is no spatial structure in the evolution morphology. For larger values of *n*, we see the emergence and growth of domains of up-spin (marked in black) and down-spin (unmarked). These domains interact and annihilate, resulting in coarsening of the characteristic length scale, and therefore, domain patterns at different times look statistically similar, apart from a global change of scale. The domain size at a fixed time (e.g., *t* = 500) is smaller for larger values of *n* (short interactions). Spontaneous and simultaneous connections among near and distant neurons is an inherent property underlying brain functionality, as the domain formed by long-range interactions grow quickly in time as compared to short range explains for compensating distance with time.

**Fig 3:**
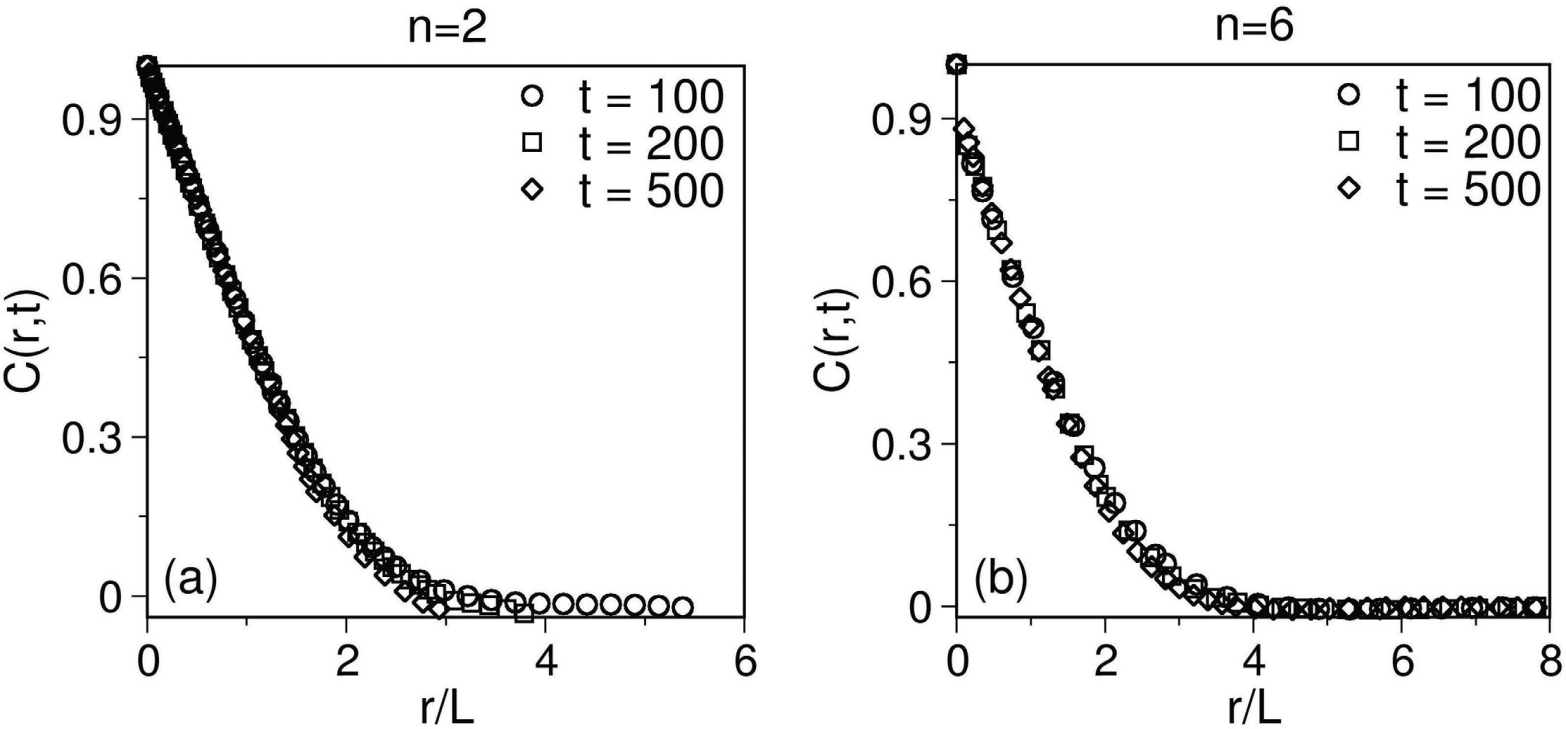
**A** Scaling plot of *C*(*r*, *t*) vs. *r*/*L* for a phase ordering dynamics in *d* = 2 for *n* = 2. The data sets (for *t* = 100, 200, 500) collapse onto a single master curve. **B** Similar plot for *n* = 6.

In Fig 3, we show a scaling plot of the correlation function, defined in Eq (11). We plot *C*(*r*, *t*) as a function of the scaled distance *r*/*L* at three time instants, as indicated. Fig 3A corresponds to *n* = 2, and Fig 3B shows data for *n* = 6. The dynamics of the spins (neurons) in terms of correlation function and structure factor at different time points has shown a perfect congruence with each other witnessing the universality in their behaviour as well as confirming the validity of dynamical scaling.

**Fig 4:**
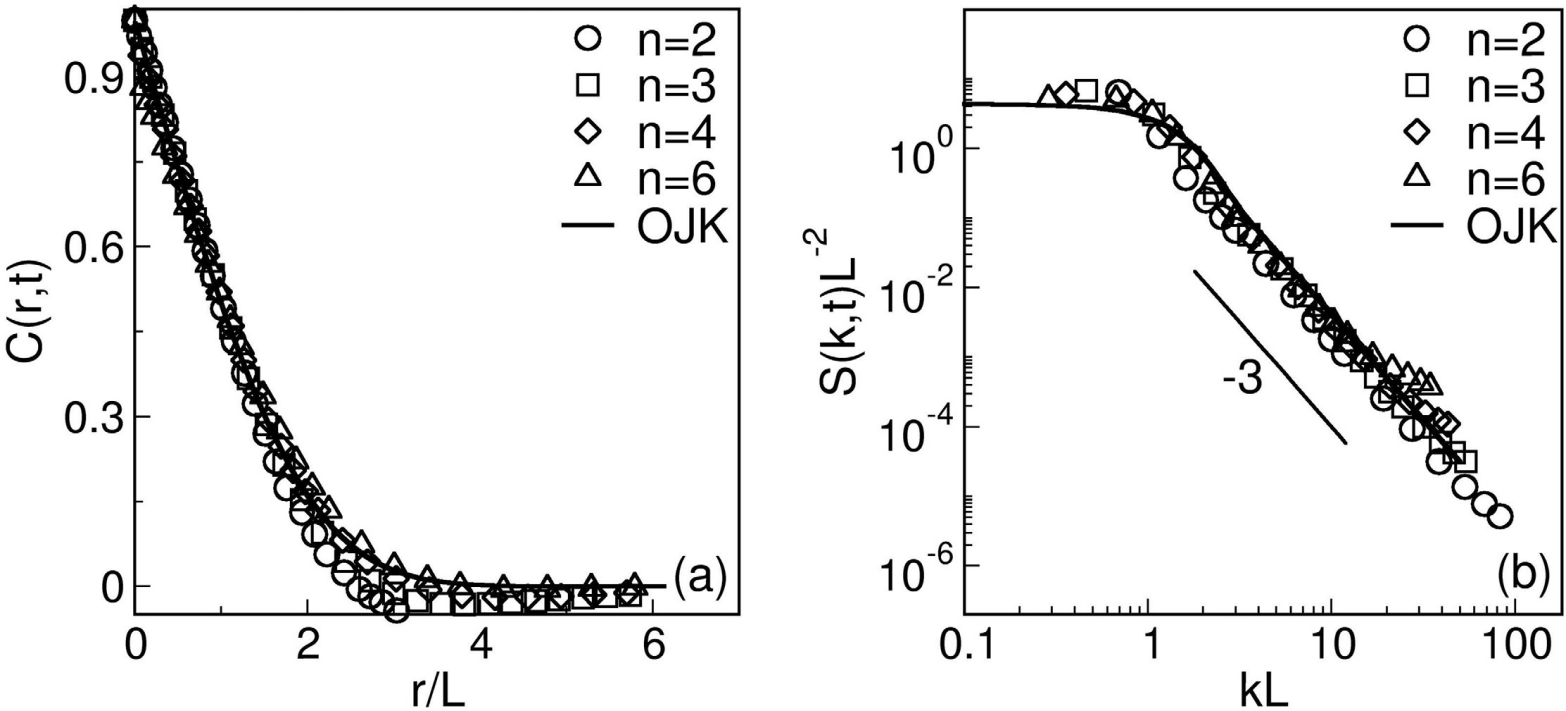
**A** Plot of *C*(*r*, *t*) vs. *r*/*L* at *t* = 500 for *n* = 2, 3, 4, 6. **B** Plot of *S*(*k*, *t*)*L* ^−2^ vs. *kL*, corresponding to the data sets in **A**. The reasonably good data collapse shows that the scaling functions do not depend on the interaction range. The solid line denotes the OJK function in Eq (15) [45].

Let us next discuss whether the evolution morphology depends on the range of the interaction characterized by *n*. Fig 4 shows a comparison of the scaling functions for four different *n* values (*n* = 2, 3, 4, 6) at a time *t* = 500, when the system is already in the scaling regime. In Fig 4A, we plot the scaled correlation functions. The reasonably good data collapse suggests that the scaling functions do not depend on the interaction range. The solid line in Fig 4A denotes the analytical result due to Ohta et al. (OJK) [45], who studied ordering dynamics in a ferromagnet. The OJK function is

$$C\left(r,t\right)=\frac{2}{\pi }\sin^{-1}\left(e^{-r^{2}/L^{2}}\right).$$ (15)

(The corresponding result for the case with vector order parameter has been obtained by Bray and Puri [46].) Our correlation-function data is in excellent agreement with the OJK function, showing that the phase ordering dynamics for *n* < 4 lie in the same dynamical universality class as that for *n* > 4. In Fig 4B, we plot the scaled structure factor [*L* ^−2^ *S*(*k*, *t*) vs. *kL*] for the same time as in Fig 4A. Again, the data sets collapse neatly onto a single master curve, confirming the scaling form in Eq (14). The scaling function is in excellent agreement with the corresponding OJK function. Notice that the structure factor, for large values of *k*, follows the well-known Porod’s law, *S*(*k*, *t*) ∼ *k* ^−(*d*+1)^, which results from scattering off sharp interfaces [47, 48]. The scaled correlation function and structure factor, in congruence with scale free behaviour of functional brain networks [49] depicts the universality of the interaction mechanism in both short and long range interactions in brain.

**Fig 5:**
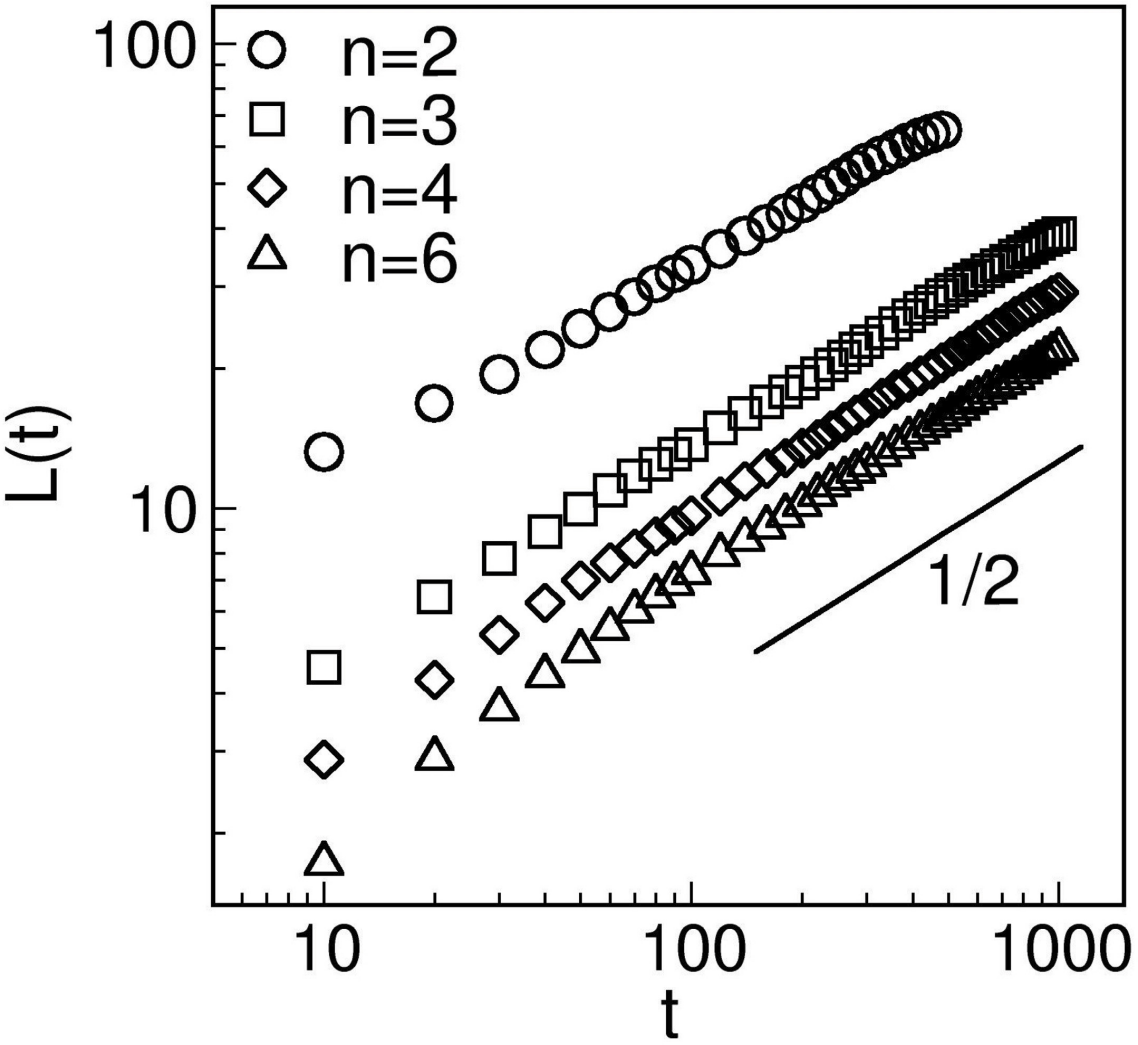
Time-dependence of the characteristic length scale *L*(*t*) for *n* = 2, 3, 4, 6, plotted on a log-log scale. The lines of slope 1/2 indicates the power-law growth regimes expected for phase ordering in *d* = 2 ferromagnetic system.

In Fig 5, we turn our attention to the time-dependence of the domain size. We plot *L*(*t*) vs. *t* on a log-log scale for *n* = 2, 3, 4 and 6. Here, the data sets are consistent with the *Cahn-Allen* growth law, *L*(*t*) ∼ *t* ^1/2^–there is no sign of a crossover in the growth law at *n* = 4, as predicted by Bray [50]. Bray has used the renormalization group (RG) approach to study ordering dynamics with long-ranged interactions of the form *r* ^*d*+*σ*^ with 0 < *σ* < 2. In our case, *d* = 2 and *σ* = *n* − 2. Bray argues that the long-ranged interactions are relevant for 0 < *σ* < 2 or 2 < *n* < 4, and irrelevant for *n* > 4. The corresponding growth law is

$$L\left(t\right)∼\{\begin{array}{cc} t^{1/(n-2)}\; & \text{for}\;2<n<4,\\ t^{1/2}\; & \text{for}\;n>4, \end{array}$$

with possible logarithmic corrections. As we can see that our numerical results are not consistent with this prediction. The only difference as *n* is varied is that we have faster growth (higher prefactors) for smaller *n*, corresponding to more long-ranged interactions. The fast dynamics of long-range interactions signifies the path of information processing and neuronal connections in the brain. The longer persistance of long-ranged neural connections could give sense to clustering behaviour of neural circuitry, specifically during learning of a specific task, new synaptic connections tend to form in vicinity of old connections related to that task [51] making it more robust. Convincing to the fact that re-learning help us to memorize things for longer duration.

**Fig 6:**
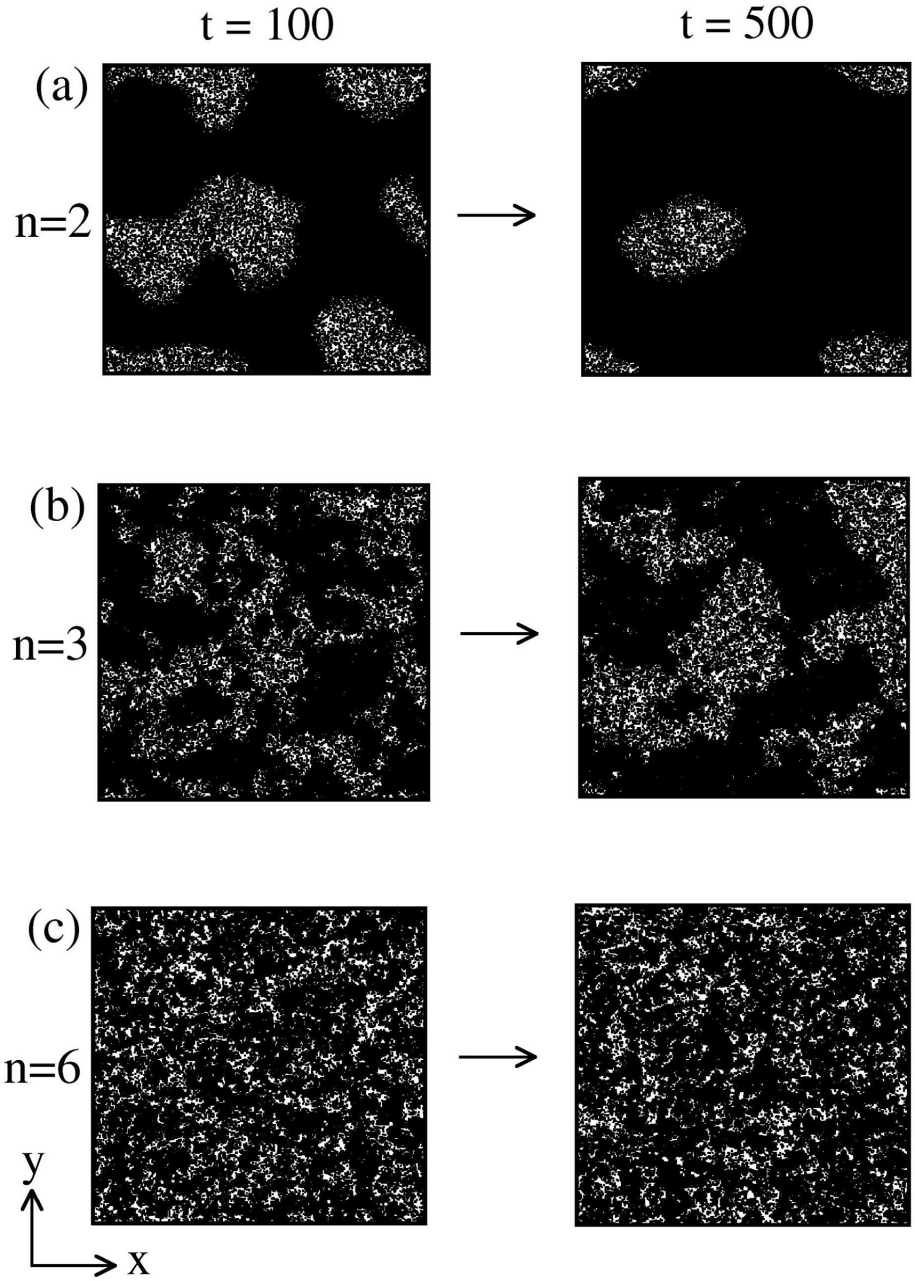
Evolution snapshots of phase ordering systems in *d* = 2 for *n* = 2, 3, and 6. The system is quenched at *T* ≃ *T*_*c*_. The MC simulations are described in the text.

In Fig 6, we show the evolution of the order parameter (*m*) near critical temperature (*T* ≃ *T*_*c*_) from a disordered initial state (*m* = 0) for *n* = 2, 3, and 6 respectively. At higher temperature below *T*_*c*_ we observe large fluctuations in the evolution patterns with very small global ordering; instead of picking one of the up-spin, down-spin, or zero order parameter states, the system near *T*_*c*_ is a kind of fractal blend of all three [52]. Giving an insight that brain code information in the form of pattern of neurons activated. For example, in visual cortex a particular information signal will activate a bunch of neurons related to that code of information and leads to recognition of an object [53]. However the cluster size is larger for smaller *n*. Recall that at *T* = 1 (≪ *T*_*c*_), thermal energy (*k*_*B*_ *T*) of the system is low, thus spins try to obtain minimal energy by forming domains with a global ordering: *m* = +1 or -1.

**Fig 7:**
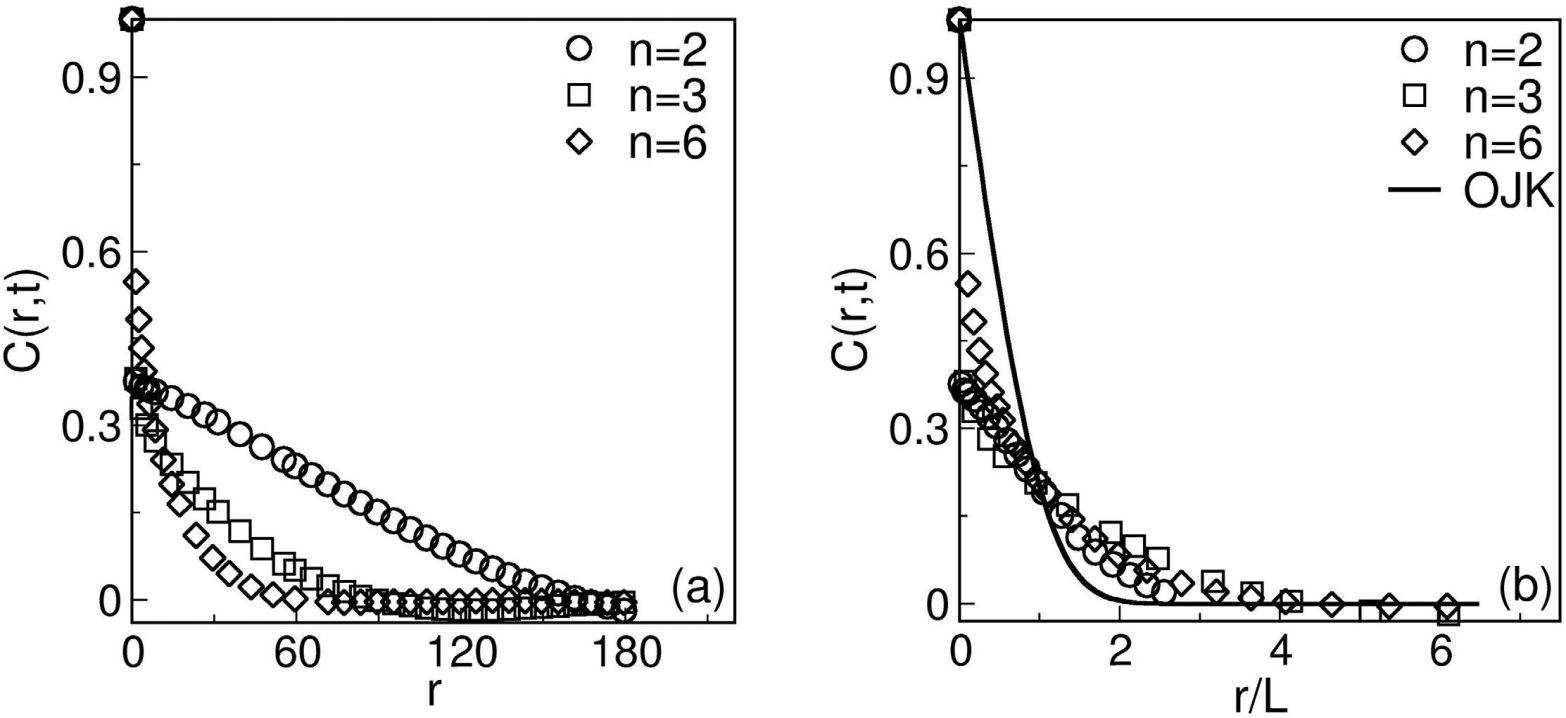
**A** Plot of *C*(*r*, *t*) vs. *r* for *n* = 2, 3, 6 at *t* = 500 MCS. **B** Scaling plot of *C*(*r*, *t*) vs. *r*/*L*, corresponding to the data sets in **A**. The solid line denotes the OJK function for the scaled correlation function in Eq (14).

Finally, in Fig 7A, we show the plot of correlation function [*C*(*r*, *t*) vs. *r*] corresponding to the evolution shown in Fig 6 at *t* = 500. Note that the decay range of the correlation function is larger for long-ranged interactions. Fig 7B shows the scaling plot of the data sets in Fig 7A. A reasonable data collapse confirms the dynamical scaling and clarifies that the system for each interaction range belongs to the same dynamical universality class.

## Summary and Discussion

Let us conclude this paper with a summary and discussion of the results presented here. We study the effect of interaction range on the morphology of neuron activity pattern. Several previous works on ising model has calculated nearest neighbor interaction at different temperature and found that it could depict the functional brain activity at criticality [21, 27, 54]. We have simulated a system of 2-D non- conserved ising model, far from equillibrium with Glauber dynamics considering a parameter that controls the coupling strength over interaction range and obtained patterns of neural activity represented as a domain showing the modularity of functional brain networks at far below and near criticality. The long-ranged and short-ranged interaction of neurons could be the main basis of how brain performs complicated functions at fundamental level. With this prior knowledge, we have studied the effect of interaction range on the morphology of the domains obtained, considered as neuron activity patterns. We analyzed the system at far from criticality (*T* = 1) and near criticality (*T* ≃ *Tc*) and obtained neuron activity patterns which in general implies that the dynamics of long- range interaction outrace the short range. As shown by our simulation results both short and long ranged interactions exhibits similar dynamics over time domain which makes its analogy with neuronal interactions flexible. We have studied the dynamics of long and short range interactions separately at fixed temperature and concluded that they follow similar kinetics when scaled, however appear different in the time domain. We anticipated that emergence of fast and slow kinetics of long and short ranged cases could imitate the wiring and rewiring of neurons for relay information transfer and topology of functioning of modules in brain. Neuron activities as well as wiring and rewiring of the neurons in the network subjected to heat bath depend on the range of interaction which are reflected in the dependence of critical temperature *T*_*c*_ and magnetization on *n*. The domain sizes of the neurons (spins) in short range interaction at far-lower critical temperature are smaller; some are isolated and numbers are more as compared to long ranged interaction for any time domain, showing their fast dynamics. However, the domain dynamics both in short and long ranged interaction system is quite different as compared to far-higher critical temperature dynamics due to emergence of more randomness in the domain organization in the system. This leads to the change in domain growth laws of the neurons in short and long ranged interaction in the system. As the system approches near critical temperature, the domain pattern formation of interaction exhibits fractal kind of behaviour that could portray similar functionality of different modules. Evolution snapshots of the system for distinct interaction range (coupling strength) at near criticality has revealed fractal nature with pattern formation signifying strong correlation to brain dynamics [53], however, power law behaviour of the characteristic domain length scale further establishes the fact. The correlation of neurons (spins) decays much faster in short ranged interaction as compared to long ranged, but it scales with *r*/*L* showing the universality of neuron interactions in brain. Thus, study of this simple system has lead us to the conjecture that the system of neurons undergoes second order phase transition near criticality and forms a pattern of active neurons performing a specific task upon receiving a signal or we can say that a signal/stimulus might take the system towards criticality [13].

Given the current focus on the biological network and their functionality, we hope that this paper will motivate fresh interest in the evolution dynamics and morphology of active and passive neurons. These kinetic processes play an important role in determining the functionality of brain. We emphasize that one can gain a good understanding of the relevant neuron dynamics (wiring and rewiring inside the brain network) from simple coarse-grained models of the type discussed here. This model could correctly anticipate interconnected neuron kinetics involved in functions like cognition, behaviour, thoughts, perception etc. One important conclusion from this study could be that when brain receives a signal it gets transformed from a random system of neurons, undergoes second-order phase transition, and turns out into an ordered sets of firing neurons. This could possibly correspond to emergence of functional modules in brain, needs more investigation. Thus it is an attempt to predict and an outlook to understand the functionalities of the brain.
